# Aneurysms in Colic

**Published:** 1892-06

**Authors:** 


### ANEURYSMS IN COLIC.

Translated by C. A. Cary, B. S., D. V. M.

Among the internal diseases of farm animals, colic in the horse plays the principal role ; also, it makes up the majority of the diseases in the hospital of the veterinarian. It is now apparent that this disease occurs more frequently in the summer months than in the winter, and consequently, at that time, brings about more losses. Also, the relative causes are more numerous and various in the warm season, since the weather influences the variations in the temperature of the air, the changes in the food and the excessive work call forth more diseases ; so, in the dissection of colic-lost horses, the alterations in the trunk vessels of the intestinal arteries point yet to other disease factors. One also observes the more frequent appearance of colic in summer, when in ætiological consideration the relation of food and work come not essentially into question. The preponderance of deaths at this time of year gives occasion to investigate the cause and also have explained its importance for correct treatment. Repeatedly have I examined, by dissection, colic-lost horses, and, of course, I directed my attention to the questions : Are aneurysms produced by nematodes, and do the larva migrate during a certain time of the year?

During the summer months of 1891 I had opportunity to make autopsies on twelve colic-lost horses, and nine of them died from contraction, inflammation and degeneration of the anterior mesenteric artery! The statistics of Bolinger will show that three-fourths of the deaths by colic are caused by worm aneurysms. I am of the opinion that all nine cases were worm aneurysms, although I could discover only five living specimens of *sclerostomum armatum* by separation and excision of every prominently degenerated mesentery blood vessel. The supposition that the aneurysms occur from the tearing of the vessel wall by the hanging of the loaded intestinal folds on the arterial trunks was not conclusive, since it is not known if embolic colic happens in other animals built like the horse. Bolinger placed the worm aneurysms in the foreground, and I agree entirely with him in this view. I had opportunity to observe horses for years in which every summer reappeared chronic colic that lasted five weeks, and no longer. In the meantime the healthy condition of these animals was without interruption ; finally, at the same time in summer, the disease again appeared and death demanded his victim. In the dissection of these animals I found in every one a greatly degenerated messentery blood vessel, with partial ossification ; the lumen was freshly inflamed, with nematodes therein. Surely the existence of worm colonies in this place in such cases does not infer or suppose that the palisades of worms repeatedly visit the branching places of the intestinal arteries from the aorta, but mostly in the trunk of the anterior mesenteric, seldom in the abdominal artery, other mesentery arteries or the venal arteries. The intima, after the inflammation of the preceding year, becomes newly perforated and irritated, until after repeated inflammation the lumen contracts so that the blood supply to the intestines is cut off. The sickness of the animal is well begun at the first migrations, thrombi and emboli, but death therefrom is not always brought about if the thrombus can degenerate into fat and separate, or if the vessel wall grows completely or partially together, in which last case ordinarily the immediately opposite vessel wall falls back, and by this means it opens to the blood current the possibility of re-entrance into the vessel. Also, in the course of time, a compensation, through sufficient collateral circulation, can take place. One may account for the frequent occurrence of colic in June, July and August by giving special attention and consideration to the fact that at such times the larva migrate, after a short stay in a known place, after that cause the injury, and during these times the larva develop into adult species, then again seek the colon and cæcum in order to live there. By means of trepan-shaped boring arms these worms are able to quickly wander out and in. In the warm time of the year the embryos escape as eggs in the fæces, to become taken up occasionally as free nematodes, with the food and drinking water of horses. Thus we observe the appearance of colic more in warm moist weather more than in dry cold seasons.

Bollinger was right when he wrote: Most cases of colic in horses are caused by the larva of the armed palisade worms. The worms are absent only in winter in old aneurysms. To prove my assertions, I have commanded only my observations, but it would be desired if investigations were otherwise conducted.—*From Berliner Thierärztliche Wochenschrift.*

The translator of the foregoing article observed two cases of worm aneurysms, in 1891, at Brookings, South Dakota.

The first case was in a two-year-old colt that died June 23, of tetanus, resulting from castration. The aneurysm was in the anterior mesenteric artery; living nematodes (*sclerostomum armatum*) were present. As far as known the colt was never troubled with colic. The second case was in a deformed colt, about 18 months old, that was used for dissecting purposes. This colt was destroyed on October 18th, and the aneurysm was found about two inches from the anterior mesenteric artery, in one of the branches that run to the small intestines. Living nematodes were also found in this aneurysm. The colt was never affected by colic. A little attention on the part of veterinarians would soon accumulate sufficient facts to make this subject clear—prove or disprove the theory. The examination for aneurysms may be made by severing the mesentery close to the intestines and then removing the posterior aorta from the diaphragm to its terminals, being careful not to tear the small mesentery arteries from their trunks; then a little care in tracing the course of the mesenteric arteries will soon determine the presence or absence of aneurysms. It is not necessary, as some have asserted, to inject plaster of paris or any injecting fluids into the vascular system in order to detect aneurysms.

